# Trends in cancer-free working life expectancy based on health insurance data from Germany–Is the increase as strong as in working life expectancy?

**DOI:** 10.1371/journal.pone.0288210

**Published:** 2023-07-26

**Authors:** Fabian Tetzlaff, Enno Nowossadeck, Jelena Epping, Vanessa di Lego, Magdalena Muszynska-Spielauer, Johannes Beller, Stefanie Sperlich, Juliane Tetzlaff

**Affiliations:** 1 Medical Sociology Unit, Hannover Medical School, Hanover, Germany; 2 Division of Social Determinants of Health, Robert Koch-Institute, Berlin, Germany; 3 Comprehensive Cancer Center Hannover, Hannover Medical School, Hanover, Germany; 4 Wittgenstein Centre for Demography and Global Human Capital (IIASA, OeAW, Univ. Vienna), Vienna, Austria; 5 Vienna Institute of Demography, Austrian Academy of Sciences, Vienna, Austria; Tampere University, FINLAND

## Abstract

**Background:**

Against the backdrop of population ageing, governments are facing the need to raise the statutory retirement age. In this context, the question arises whether these extra years added to working life would be spent in good health. As cancer represents a main contributor to premature retirement this study focuses on time trends and educational inequalities in cancer-free working life expectancy (WLE).

**Methods:**

The analyses are based on the data of a large German health insurer covering annually about 2 million individuals. Cancer-free WLE is calculated based on multistate life tables and reported for three periods: 2006–2008, 2011–2013, and 2016–2018. Educational inequalities in 2011–2013 were assessed by two educational levels (8 to 11 years and 12 to 13 years of schooling).

**Results:**

While labour force participation increased, cancer incidence rates decreased over time. Cancer-free WLE at age 18 increased by 2.5 years in men and 6.3 years in women (age 50: 1.3 years in men, 2.4 years in women) between the first and third period while increases in WLE after a cancer diagnosis remained limited. Furthermore, educational inequalities are substantial, with lower groups having lower cancer-free WLE. The proportion of cancer-free WLE in total WLE remained constant in women and younger men, while it decreased in men at higher working age.

**Conclusion:**

The increase in WLE is accompanied by an increase in cancer-free WLE. However, the subgroups considered have not benefitted equally from this positive development. Among men at higher working age, WLE increased at a faster pace than cancer-free WLE. Particular attention should be paid to individuals with lower education and older men, as the general level and time trends in cancer-free WLE are less favourable.

## Introduction

Population ageing is a key challenge of the 21st Century [1]. Although increasing life expectancy is a remarkable achievement in human history, population ageing is challenging social security systems, especially in high-income countries. For example, the German government raises the statutory retirement age from 65 to 67 since 2012 until 2030 [2]. A further increase in the retirement age is currently subject of a controversial debate in Germany. Cancer represents one of the main contributors to premature retirement from working life [3, 4]. Studies have shown that individuals with a lower socioeconomic status (SES) have higher risks of incidence, a reduced probability of survival after incidence, a higher mortality, and consequently a lower life expectancy and working life expectancy (WLE) compared to individuals with higher incomes, educational degrees, and occupational positions [5–14]. By extending of the working life and increasing the statutory retirement age towards 67, the intended upper limit of working age is approaching the average age of onset of various cancer types. For most cancers, the average age of onset is approximately around 70 years and above [15]. Moreover, a recent study showed that a substantial number of years of life are lost due to cancer in Germany [16]. Against this background, the question arises as to whether people would be able to remain in the labour force up to even higher ages in the future from a health perspective. The aim of the study is to contribute to the current research by analysing time trends in cancer-free working life years and comparing its developments to those of working life expectancy altogether. Additionally, educational differences in cancer-free WLE will be considered.

International studies reported increasing WLE for both sexes in several European countries, with increases being usually stronger in women than in men [6, 17–21]. Furthermore, clear educational inequalities in the remaining years in labour force were found [6, 17–19, 21]. In Germany, there are only two studies so far that have examined educational inequalities in WLE. The studies show that individuals with lower education have lower WLE and conclude that SES factors are strong drivers of inequalities in WLE [21, 22].

Health can be measured in several way. Most studies on HWLE so far focused on more general health outcomes, such as self-rated health or disability, for which the diseases causing poor health are usually unknown. The advantage of focusing on specific diseases is that the studies can help to identify disease-specific prevention needs in the working-age population. The need for research that focuses on the development of WLE in terms of specific chronic diseases has only recently been identified and very few studies have been published so far, which reported that the gains in the WLE are accompanied by additional unhealthy working years [18]. Overall, chronic cardiovascular diseases, diseases of the skeletal system and diabetes are most often responsible for life years spent working and in ill health. Oncological diseases also contribute to a non-negligible share of unhealthy working lifespan, with women working longer after the onset of oncological diseases than men [18]. However, studies which examine the development of healthy WLE in terms of specific chronic diseases among the German population are lacking. Since cancer represents in most cases a fatal diagnosis, which leads to long-standing impaired health and often ends working life, our study aims to step into this gap by investigating time trends in cancer-free WLE between 2006 and 2018 based on German statutory health insurance data. An additional emphasis will be given to educational differences in cancer-free WLE. The study addresses the following research questions:

1. How did cancer-free working life expectancy develop over time? Did the developments differ between the sexes?
2. Are there educational inequalities in cancer-free working life expectancy?
3. How did cancer-free working life expectancy develop compared to total working life expectancy over time?

## Methods

### Data

Our analyses are based on longitudinal claims data, i.e., on routinely collected data from a large German statutory health insurance provider (Allgemeine Ortskrankenkasse Niedersachsen), which covers about 37% of the inhabitants of the federal state of Lower Saxony [23]. The data were fully anonymised before we accessed them. The use of this sort of data for scientific purposes is regulated by federal law. The data protection officer of the Statutory Local Health Insurance of Lower Saxony has approved its use. In addition to diagnosis codes based on the International Statistical Classification of Diseases and Related Health Problems (ICD-10GM), medical treatments and mortality, the insurance histories of insured persons also contain information on sociodemographic (e.g. educational level) and labour force characteristics (e.g. employed, unemployed, retired, etc.) [21]. As shown in previous studies, the data are comparable to the total German population in terms of sex and age distributions. However, the socioeconomic composition differs from the total population, as individuals with higher socioeconomic positions are underrepresented [23]. In order to identify possible potentials in working beyond the official retirement age and to draw a detailed picture of the entire working life, we used the data of all insured individuals aged 18 to 69.

### Definition of labour force

The concept of labour force of the International Labour Force Organization (ILO) was used to operationalise labour force and non-labour force [24]. Following this definition, individuals in paid work and unemployed individuals are part of the labour force as well as employed individuals currently absent from work due to illness, parental leave or further vocational training. The non-labour force includes retired and studying individuals, as well as individuals who are not part of the labour force for other reasons. Based on the health insurance data, it is possible to largely follow the ILO labour force concept. A detailed description of how the employment histories can be defined in the data and a comprehensive discussion of the methods can be found in Tetzlaff et al. [21].

### Definition of incidence

In order to identify cancer incident cases in our data, we used the ICD-10GM diagnosis coded within the insurance histories. All cancer-related codes (C00-C98) were used in the analysis. Since the paper focuses on primary malignant neoplasm, non-melanoma skin cancer (C44) and tumours of non-specific, secondary and undefined locations (C77-C79) were excluded. To identify incident cases and secure the validity of the incident diagnosis, a multi-step approach was used, in which the first cancer diagnosis was validated by using 1-year look back periods and the minimum-2 quarters criterion for outpatient diagnoses. A description of the operationalisation of incident cases can be found in detail in an earlier study [25]. To analyse time trends in cancer-free working life years, the data were divided into three periods: 2006 to 2008, 2011 to 2013, and 2016 to 2018.

### Definition of educational level

To analyse educational inequalities in cancer-free working life years, we used the information on school leaving certificates. To facilitate international comparability, the minimum period of schooling required to obtain the certificates was used as an indicator of educational level. The individuals were categorised into two groups: 8 to 11 years of schooling (lower educational level) and 12 and more years of schooling (higher educational level).

In Germany, employers are legally obliged to report on school leaving certificates to the statutory health insurance provider. This means, that this information is often only available for employed persons. Therefore, the proportion of missing values is rather high among the non-labour force (57%) compared to employed individuals (27%). For longer time series data it is possible to transfer this information from previous or following insurance episodes in case the individual is currently not employed. Therefore, educational information is usually available in the data if a person was employed at any time between 2005 and 2018 [21]. However, educational information is often missing for retired individuals at the beginning and for younger individuals not yet employed at the end of the observation period. Due to this unequal distribution of missing values over time, educational inequalities in the cancer-free WLE were analysed for the middle period (2011 to 2013) only [21]. Since the group of individuals without information on education is rather heterogeneous, the analyses on inequalities in cancer-free WLE are limited to the lower and higher educational group. To reduce the share of missing values, we used the same imputation strategies of transferring the education information from one spouse to the other and from parents to their to their child who were not (yet) employed in case of missing information that have already been applied in previous studies based on the same dataset [21, 26]. Using these strategies, the share of missing values on education was reduced to 41% in the non-labour force.

### Statistical analysis

**Fig 1:**
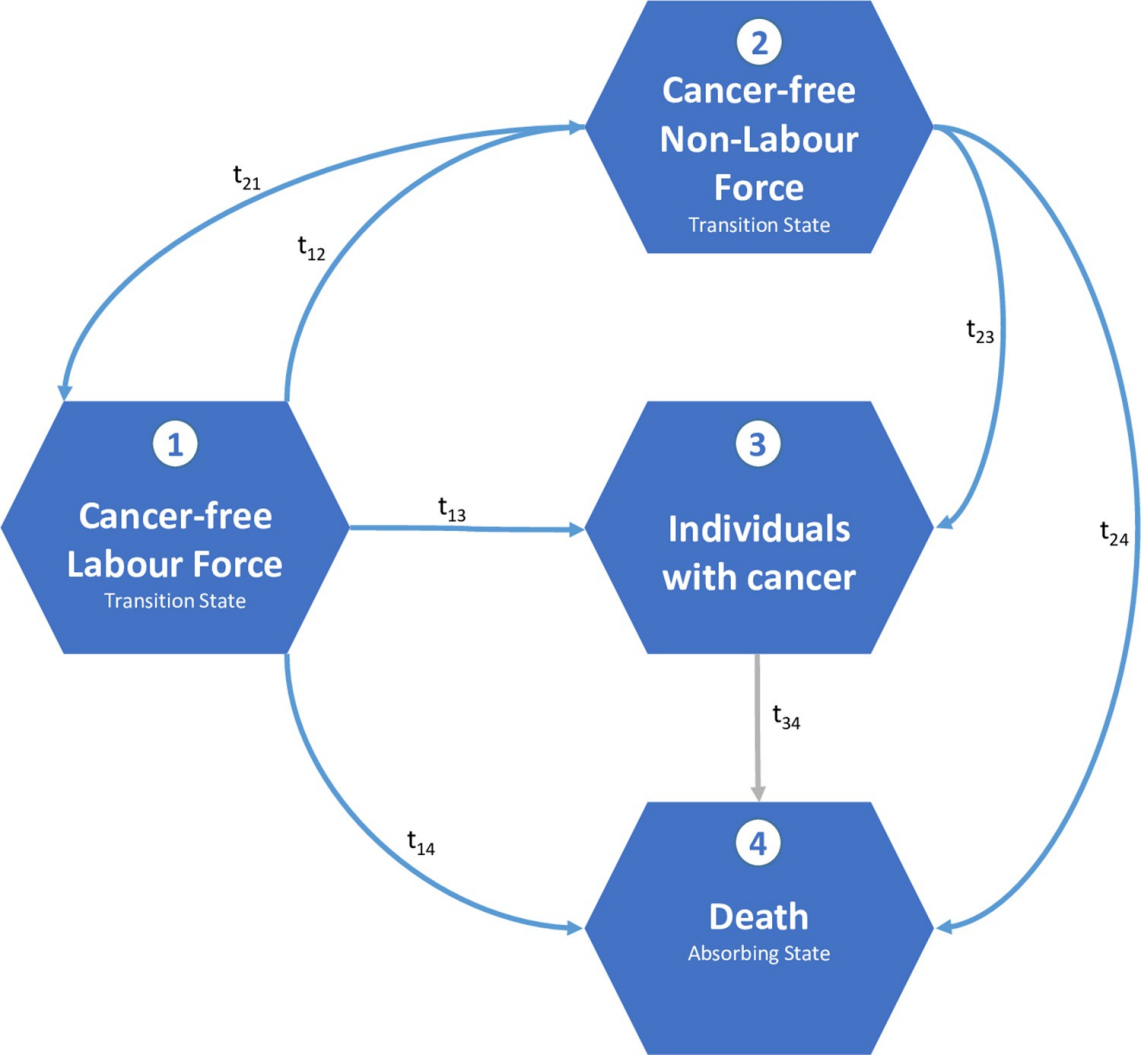
Four-state illness-death model (cancer-free labour force, cancer-free non-labour force, individuals with cancer, and death) and the corresponding transitions.

The analysis of cancer-free working life expectancy is based on a four-stage illness-death model with seven possible transitions between the states (Fig 1).

The model contains two transition states (cancer-free labour force, cancer-free non-labour force) and the state “cancer diseased” and “death”. With the fist cancer diagnosis, the individuals move to “cancer diseased” health state and no return to a “cancer free” state is possible. The most frequent transitions are those from the cancer-free labour force to the cancer-free non-labour force (t_12_) and vice versa (t_21_). Moreover, the model considers that individuals of every living state may be diagnosed with cancer (cancer incidence t_13_ and t_23_) or can die with or without cancer (death rates t_14_, t_24_, t_34_). For the calculation of the cancer-free WLE, however, the transition t_34_ is not considered since cancer-free WLE is defined as the length of working life spent prior to the first diagnosis. All other observed age-specific transition rates were used as input for the multistate life table analysis [27] to estimate cancer-free WLE. The analyses were stratified by sex, period, and educational level (for the second period only, as described above). Cancer-free WLE is reported as partial life expectancy between age x and age 69 since the percentage of individuals belonging to the labour force above age 69 is very low (<1%). In order to examine the development of cancer-free WLE over time relative to total WLE, the proportion of cancer-free WLE is also reported; e.g. proportion of 100% would indicate that the entire remaining WLE is expected to spent without cancer. The 95% confidence intervals are estimated with 1000 bootstrap sample replications. The analyses were performed using Stata 17 and R 4.1.2.

## Results

The study population includes 48% men and 52% women. The number of individuals in the labour force is increasing in total as well as in both sexes across periods (Table 1 and S1 Table in S1 File). This increase is more pronounced in women than in men and is accompanied by a decrease in the number of individuals in the non-labour force. Over the entire time period, approximately 24% of the incident and 8% of the death cases occur in the labour force. Stratified for education, the majority of the study population also belongs to the labour force (Table 1).

**Table 1:** Characteristics of the study population aged 18 to 69: exposures in person-years and number of failures by type of transition, sex, and time period, as well as educational group in the period 2011–2013.

		Men					Women				
		2006–2008	2011–2013	2016–2018	lower educational level (2011–2013)	higher educational level (2011–2013)	2006–2008	2011–2013	2016–2018	lower educational level (2011–2013)	higher educational level (2011–2013)
**Labour force**	number of individuals	643,985 (27%)	771,838 (33%)	945,506 (40%)	504,626 (87%)	77,120 (13%)	471,235 (26%)	564,769 (32%)	746,431 (42%)	354,805 (81%)	85,462 (19%)
**Non-labour force**		429,516 (32%)	460,397 (34%)	464,404 (34%)	150,013 (79%)	40,657 (21%)	735,872 (34%)	738,213 (34%)	690,113 (32%)	242,076 (82%)	54,533 (18%)
**Transition t**_**12**_ cancer-free labour force → cancer-free non-labour force	events	82,067 (27%)	102,294 (33%)	123,165 (40%)	65,548 (83%)	13,770 (17%)	80,669 (27%)	98,222 (33%)	121,685 (40%)	63,471 (81%)	15,175 (19%)
	person years	1,476,191 (28%)	1,783,957 (33%)	2,085,327 (39%)	1,223,864 (89%)	158,394 (11%)	1,024,256 (26%)	1,256,090 (32%)	1,632,150 (42%)	810,062 (82%)	176,287 (18%)
**Transition t**_**13**_ cancer-free labour force → cancer incident individuals	events	6,058 (27%)	7,965 (35%)	8,414 (38%)	4,828 (93%)	348 (7%)	5,353 (27%)	6,661 (33%)	7,887 (40%)	4,055 (88%)	566 (12%)
	person years	1,510,108 (27%)	1,830,294 (33%)	2,151,849 (40%)	1,259,638 (88%)	164,291 (12%)	1,051,832 (26%)	1,294,817 (32%)	1,686,282 (42%)	838,491 (82%)	182,679 (18%)
**Transition t**_**14**_ cancer-free labour force → deceased individuals	events	3,821 (28%)	4,307 (32%)	5,311 (40%)	1,985 (96%)	89 (4%)	1,071 (27%)	1,235 (32%)	1,598 (41%)	455 (92%)	38 (8%)
	person years	1,510,011 (27%)	1,830,184 (33%)	2,151,749 (40%)	1,259,556 (88%)	164,290 (12%)	1,051,818 (26%)	1,294,796 (32%)	1,686,257 (42%)	838,475 (82%)	182,678 (18%)
**Transition t**_**21**_ cancer-free non-labour force → cancer-free labour force	events	82,378 (28%)	98,378 (34%)	111,020 (38%)	65,954 (77%)	19,984 (23%)	91,986 (28%)	112,844 (34%)	125,542 (38%)	74,288 (75%)	24,251 (25%)
	person years	892,782 (33%)	935,999 (34%)	897,745 (33%)	214,386 (77%)	63,233 (23%)	1,736,428 (35%)	1,702,231 (35%)	1,494,911 (30%)	474,540 (84%)	90,582 (16%)
**Transition t**_**23**_ cancer-free non-labour force → cancer incidence	events	21,530 (34%)	22,172 (35%)	19,516 (31%)	2,446 (95%)	119 (5%)	25,736 (36%)	24,658 (34%)	21,533 (30%)	3,107 (94%)	185 (6%)
	person years	916,675 (33%)	966,403 (34%)	926,781 (33%)	233,927 (77%)	70,266 (23%)	1,765,490 (35%)	1,738,438 (35%)	1,529,623 (30%)	498,330 (84%)	97,976 (16%)
**Transition t**_**24**_ cancer-free non-labour force → deceased individuals	events	23,819 (30%)	27,117 (34%)	29,103 (36%)	1,666 (97%)	50 (3%)	40,337 (33%)	41,813 (34%)	41,307 (33%)	914 (96%)	34 (4%)
	person years	916,642 (33%)	966,359 (34%)	926,740 (33%)	233,910 (77%)	70,266 (23%)	1,765,454 (35%)	1,738,396 (35%)	1,529,578 (30%)	498,320 (84%)	97,976 (16%)

Data: Health insurance data from the AOKN (Allgemeine Ortskrankenkasse Niedersachsen)

Note: Education: lower education (less than 12 years of schooling) higher education (more than 12 years of schooling); Cancer-free working life expectancy represents the expected number of years spent in the labour market that are free of cancer. Cancer-free working life expectancy is calculated based on the age-specific transition rates between four states: healthy labour force, healthy non-labour force, individuals with cancer, and death

Over time, a considerable decrease in cancer incidence has been observed. This is true for both sexes as well as for the labour force and the non-labour force (S1 Fig in S1 File). As expected, the incidence is higher among individuals not in the labour force than among those in the labour force. Furthermore, the gap between labour and non-labour force in cancer incidence persists over time (S2 Fig in S1 File).

### Development of cancer-free working life expectancy

**Fig 2:**
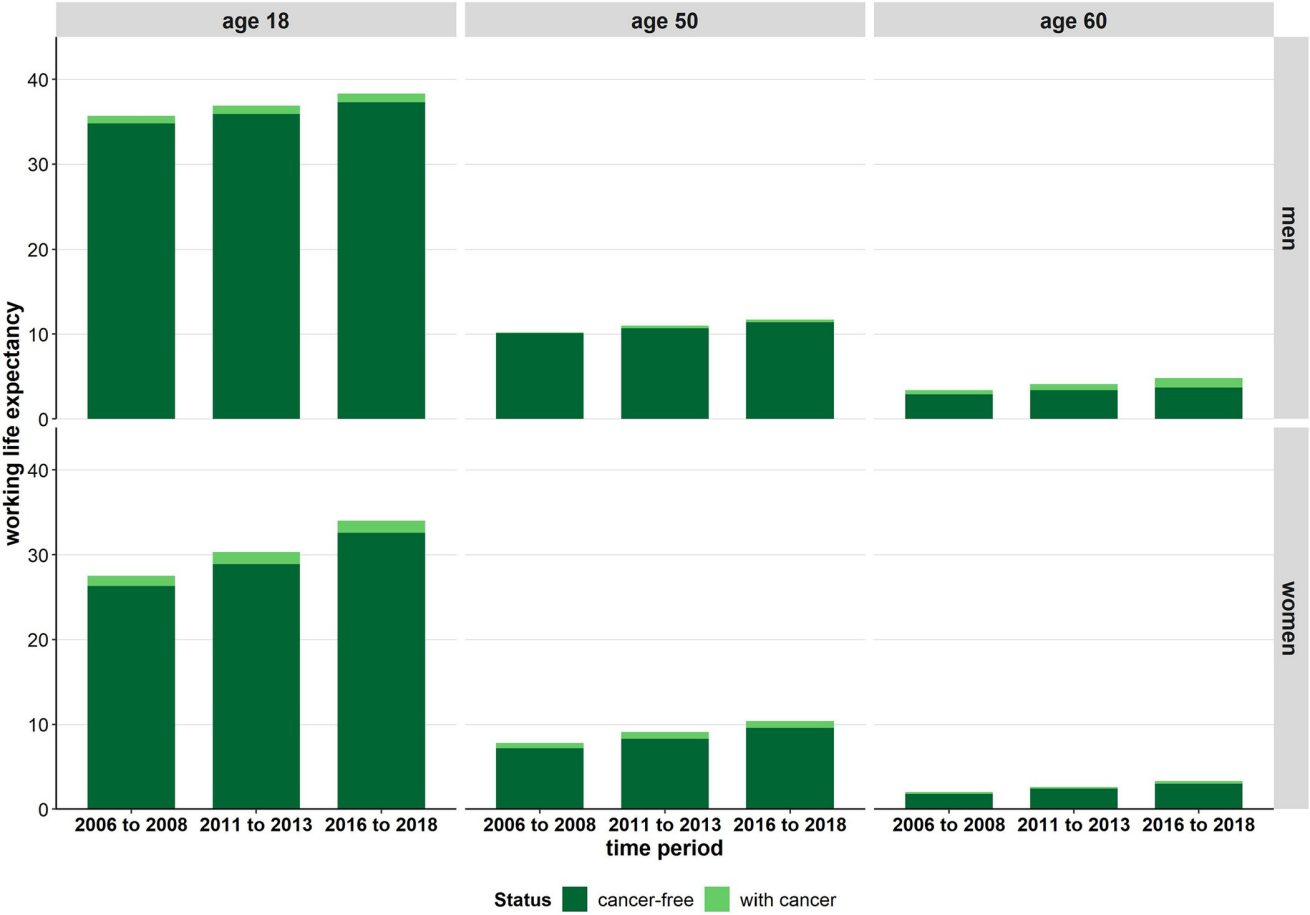
Time trend in cancer-free working life expectancy and working life expectancy after a cancer diagnosis by sex. Note: Cancer-free working life expectancy represents the expected number of years spent in the labour market that are free of cancer. Cancer-free working life expectancy and working life expectancy after a cancer diagnosis is calculated based on the age-specific transition rates between four states: healthy labour force, healthy non-labour force, individuals with cancer, and death; the time trend in cancer-free working life expectancy is statistically significant based on bootstrapped 95% confidence intervals. The time trend in working life expectancy after a cancer diagnosis is significant only in men aged 60 and above.

Cancer-free WLE (i.e. the expected number of years in the labour force that are free of cancer) in men and women clearly increased up to the higher working age (Fig 2). This development is driven by the increasing proportion of people who are in the labour force and healthy in the total working-age population (S3 Fig in S1 File). This trend is more pronounced among women (S4 Fig in S1 File). Focusing on the development of cancer-free WLE at age 18, substantial increases of 2.5 years in men and 6.3 years in women were observed between the first and the last period. These increases are also evident at higher working age (+1.3 years in men and +2.4 years in women at the age of 50, +1.0 years in men and +1.2 years in women at the age of 60) (Fig 2). However, life years spent in the labour force after a cancer diagnosis also increased slightly. These increases were most pronounced in men at higher working age. In parallel, the cancer-free non-WLE of men and women decreased, which suggests that the inactive lifetime spent free of cancer reduced over time. The declines are strongest in women, among whom the level of cancer-free non-WLE is much higher than among men (S4 Fig in S1 File).

### Educational inequalities in cancer-free working life expectancy

**Fig 3:**
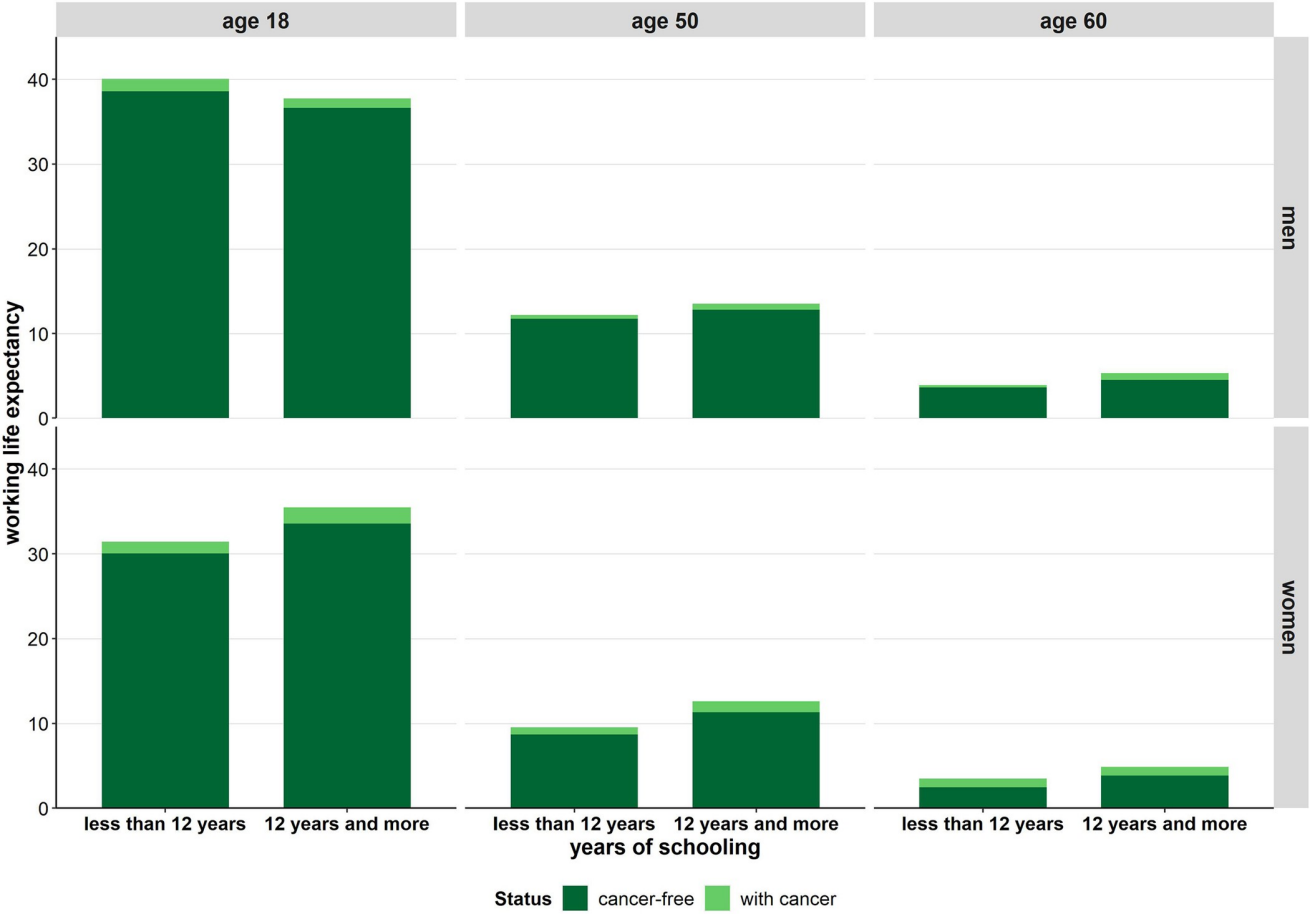
Cancer-free working life expectancy and working life expectancy after a cancer diagnosis at age 18, age 50, and age 60 by sex and education in period 2011 to 2013. Note: Cancer-free working life expectancy represents the expected number of years spent in the labour market that are free of cancer. Cancer-free working life expectancy and working life expectancy after a cancer diagnosis is calculated based on the age-specific transition rates between four states: healthy labour force, healthy non-labour force, individuals with cancer, and death; educational inequalities in cancer-free working life expectancy and working life expectancy after a cancer diagnosis (except women 60 and above) is statistically significant based on bootstrapped 95% confidence intervals.

In the second period, clear inequalities in cancer-free WLE were found between educational groups. Men aged 18 with lower educational level can expect a 1.9 years higher cancer-free WLE than men with higher educational attainments. If the focus is on age 50, higher educated men can expect more cancer-free WLE than men with lower education. This difference amounted to 1 year at age 50 and 0.9 years at age 60 (Fig 3). In women on the other hand, similar patterns were observed irrespective of the age considered. Higher educated women can expect higher cancer-free WLE than women with lower levels of education (+3.5 years at age 18, +2.6 years at age 50, and +1.3 years at age 60). However, the difference in cancer-free WLE between the educational groups increases with age and is strongest at higher working age (Fig 3). Due to their higher total WLE, the number of years spent in the labour market after a cancer diagnosis is slightly higher among men and women with higher education. This tendency is most apparent at higher working age (Fig 3).

### Development of cancer-free working life expectancy relative to total working life expectancy

**Fig 4:**
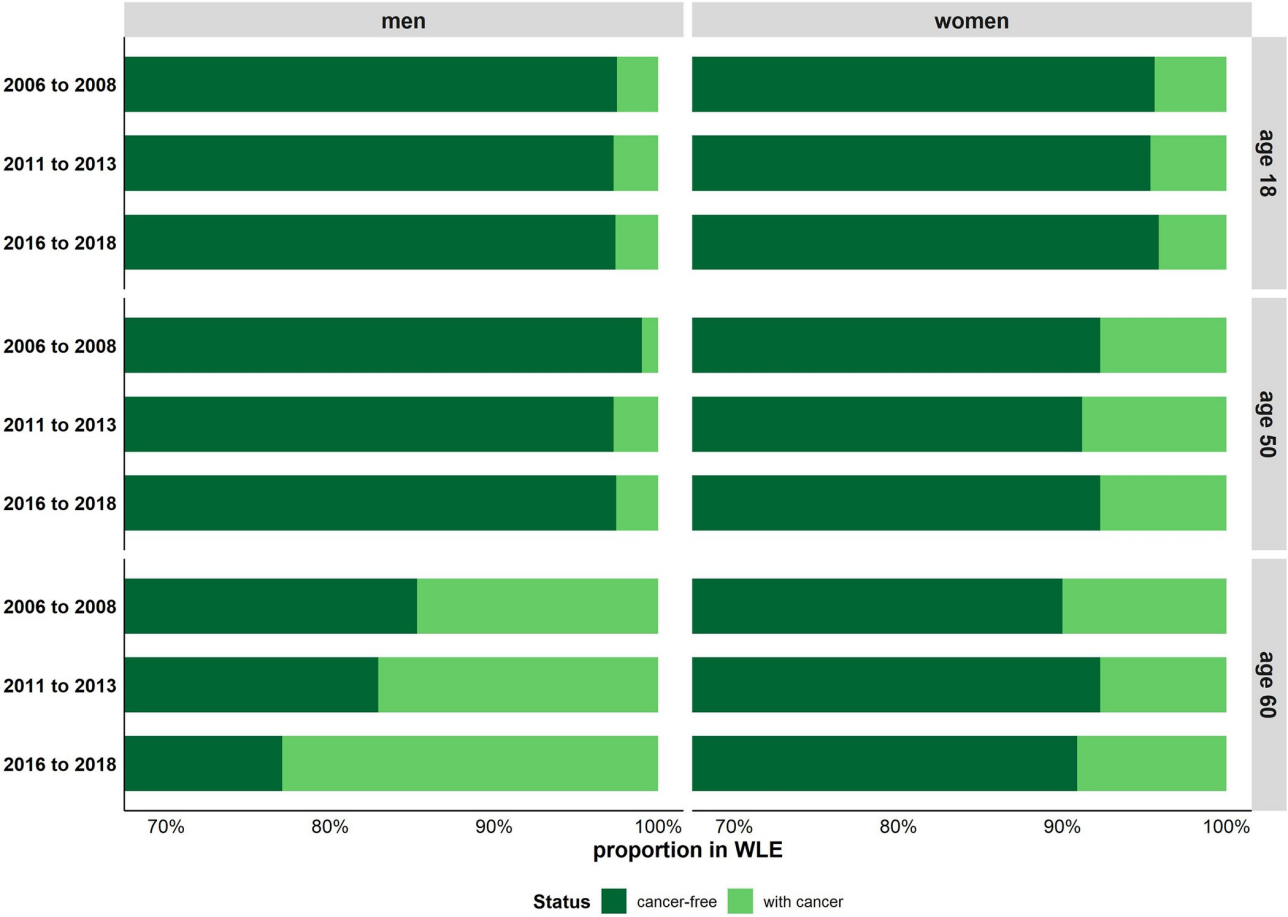
Proportion of cancer-free working life expectancy and working life expectancy after a cancer diagnosis in working life expectancy by sex and period. Note: Cancer-free working life expectancy represents the expected number of years spent in the labour market that are free of cancer. Cancer-free working life expectancy and working life expectancy after a cancer diagnosis is calculated based on the age-specific transition rates between four states: healthy labour force, healthy non-labour force, individuals with cancer, and death.

Fig 4 depicts the proportion of cancer-free WLE which makes it possible to assess the development of the cancer-free WLE relative to those of total WLE. The proportion of cancer-free WLE in total WLE remains fairly stable for men aged 18, whereas the proportion decreased for men aged 50, and even more pronounced at age 60. This decrease is due to cancer-free WLE increased at a slower pace than total WLE over time. For women the proportion of cancer-free WLE remained stable over time. The results indicate that the increases in cancer-free WLE have been strong enough to keep the relative proportion in total WLE constant, despite WLE has increased substantially (Fig 4).

## Discussion

The aim of the study was to investigate time trends and educational inequalities in cancer-free WLE in Germany. The study shows that the increases in the number of years spent in the labour force after a cancer diagnosis remained limited, although the length of working life has increased substantially over time. Moreover, the analyses reveal that men and women benefitted from the positive health trend in cancer incidence, which fostered the increase in cancer-free WLE. The study shows that the increases in WLE were accompanied by increases in cancer-free WLE that were strong enough to keep the proportion of cancer-free WLE in total WLE among women and men over time stable. An exception to that are men of older working age, for whom the proportion decreases over time indicating that the years in the labour market increased faster than cancer-free life years. This is due to the fact that the decline in cancer incidence rates is counterbalanced by the stronger increase of labour force participation, which holds especially for the age of 60 and above. Driven by higher cancer rates and earlier exits from the labour force, men and women with low education had a significantly lower cancer-free WLE than their counterparts with higher educational attainment. An exception is men with low education at a young working age, for whom the cancer-free WLE is higher because they enter the labour market earlier than men with higher education.

The debate on extending the length of working life has gained growing public attention and there is an increasing body of international research on time trends and social inequality in WLE. Our findings are consistent with previous research that has reported an increase in WLE among men and women as well as lower WLE among men and women with low socioeconomic position due to earlier exit from the labour force [5, 7, 14, 17, 19–22, 28–35]. Although cancer represents only one of several other diseases causing premature labour market exists, studies investigating trends in cancer-free WLE are vital as most cancer diagnoses represent life threating events and are associated with long periods of illness, low rates of returns to work, or death [3, 4, 36]. For total cancer, but also for the most common cancer sites, the incidence in Germany has decreased or remained more or less constant in the last decades. Two exceptions to this overall positive trend are skin cancer (both sexes) and lung cancer in women, for which incidence increased over time [15]. Furthermore, there are clear social differences in the incidence of total cancer as well as for lung, skin, stomach, kidney, bladder, and cervix cancer [13, 37, 38]. So far, there are hardly any studies on how these trends in incidence shape the trend in cancer-free life expectancy over time. A recent study based on the same health insurance data found increasing cancer-free years for total, as well as for most of the most common site-specific cancers [25]. In line with the official statistics on trends in cancer incidence rates [15], decreasing life years free of female lung cancer and skin cancer in both sexes were found [25]. However, the strong growth in cancer-free life years in terms of total cancer indicates that the potential of life time spent both, cancer-free and working, has increased over time [25].

Previous studies investigating healthy WLE mostly rely on subjective health measures (e.g. subjective health and self-reported health status [18, 20, 32, 35, 39] or self-reported disability [28]) and there are hardly any studies which investigated trends in (un-)healthy WLE with respect to specific diseases. So far only one study which reports trends in WLE affected by cancer [18]. This latter study found constant WLE affected by cancer in men and increases in women in all groups of countries considered. However, the study analysed on cancer-affected WLE while ours investigated trends in cancer-free WLE and therefore focused on developments in years lived in a healthy state. Nevertheless, our findings are in line with this study since cancer-affected WLE has increased too in our data. This becomes apparent since the proportion of cancer-free WLE did not increase over time, but remained constant or even decreased among men at higher working age. Against the backdrop of the general increase in WLE over time, this can only result from increasing numbers of life years spent working after a cancer diagnosis.

When it comes to trends in Germany, the evidence is even more limited and trends on cancer-free WLE are so far not available. Recent studies examined trends in WLE in comparison with trends in health expectancies or HWLE according to different health outcomes [19, 20]. This shows that the expansion of healthy lifespans in Germany is not limited to cancer [25], but is also found in terms of more general health indicators (e.g. years of life in good self-rated, physical or cognitive health) [19, 20]. However, clear socioeconomic inequalities in healthy WLE at higher working age were reported for several European countries, as individuals with a lower socioeconomic position have lower levels of WLE and health expectancies [19]. In line with previous research [22], we found similar patterns of socioeconomic inequalities in the length of working life which contributed strongly to the reported inequalities in cancer-free WLE. Moreover, substantial inequalities were reported in cancer-free life expectancy with individuals with lower incomes having lower health expectancies [25], which contributes to the inequalities in cancer-free WLE. The present study focused on educational inequalities in cancer-free WLE in 2011–2013 for methodological reasons. However, since large income inequalities in cancer-free life expectancy were also reported [25], future studies should investigate whether social inequalities in cancer-free WLE have widened or narrowed over time.

### Strength and limitations

In this study, routine data from a large German statutory health insurance provider were used. To our knowledge, this is the first study to examine time trends and educational inequalities in healthy WLE focusing on a specific disease using this kind of data. A major strength of the data is that the structure of the data allowed us to analyse employment histories in combination with outpatient and inpatient diagnoses, socioeconomic status and mortality. This detailed information and the high number of individuals included in the analyses allowed us to perform complex multistate.

The majority of international studies examining social inequalities or trends in healthy WLE are based on survey data (e.g. Sullivans method [19, 20, 35] or multistate life table modelling [18, 28, 39–41]). An advantage of using health insurance data is that the data are unaffected by health related-response biases, which may occur in survey data because of non-response or drop out due to health problems. Accordingly, healthy WLE based on survey data may be overestimated, particularly if severe diseases are considered. Health insurance data include all insured individuals, regardless of their current health status. Furthermore, the data are comparable to the total German population in terms of the proportion of employees subject to social insurance contributions [23] while individuals with lower SES are overrepresented [23]. A limitation of the data is that WLEs can expected to be lower than in the general population since higher WLE is associated with higher socioeconomic position.

Our study reports cancer-free WLE based on the ILO labour force concept. Accordingly, periods spent in employment and unemployment contribute to WLE. A strength of this approach is that WLE depicts the potential years in employment rather than the number of years actually spent in paid work [21, 24], which can be considered a strength when analyzing the potential of further development of WLE from a health perspective. Furthermore, our approach allows us to compare our results with those of previous studies [17, 19, 21], as well as with the official statistics on WLE from Eurostat [42] for Germany. As expected, the general level of total WLE is lower in this study. However, the time trends found in our study are very close to those of WLE reported by Eurostat [42]. Another advantage of the data is that mortality information of each insured individual is available which allowed us to consider the effect of mortality in the calculation of cancer-free WLE. Due to the increasing effect of mortality with age, many studies based on data without information on mortality had to set the upper age limit to age groups close or even before the statutory retirement. This implies that years in labour above this limit have to be ignored and the actual WLE in the total population is underestimated. A strength of the health insurance data used in this study is that it allowed us to calculate WLE between age 18 and 69 and account for the increases in labour force participation up to the very high working age.

As described in a previous methodological study on the use of routine data to calculate WLE [21], a limitation of the study is that it was not possible for us to analyse time trends in educational inequalities in cancer-free WLE. However, the theoretical assumptions and imputation strategies described in this previous study enabled us to analyse educational inequalities in WLE in the period 2011–2013. It cannot be ruled out that the educational information imputed differ in some cases differ from the true individual’s education. Regardless of theoretical and methodological considerations, similar patterns of educational differences in WLE and healthy WLE have also been reported in international studies (e.g. [18, 19, 22, 28]), which supports our theoretical and methodological approach [21] to analyse educational inequalities in healthy WLE based on health insurance data.

## Conclusion

Cancer represents one of the leading causes of premature retirement and unhealthy working lifespan. Therefore, the development of cancer-free WLE should be given special consideration when designing policies aimed at, for example, raising the retirement age. The study shows that cancer-free WLE increased clearly over time. This increase was stronger in women than in men. However, not everyone benefitted equally from this positive development. While this increase among women has been strong enough to keep pace with the extension of working lives, the increase in WLE among older men is associated with a rising portion of economically active years after a cancer diagnosis. Prevention strategies specifically tailored to the needs of the working population could help reduce cancer incidence in the labour force even further and contribute to more healthy years in the labour market. Particular attention should be paid to women and individuals with low education, for whom the general level of cancer-free WLE is particularly low in absolute terms, and men at higher working-age, as their share of cancer-free WLE decreased over time.

## Supporting information

S1 File (PDF)
