# Supplementary material for: Trends in cancer-free working life expectancy based on health insurance data from Germany–Is the increase as strong as in working life expectancy?
Source: PLoS One. 2023 Jul 26;18(7):e0288210. doi: 10.1371/journal.pone.0288210 (PMC10370751; doi:10.1371/journal.pone.0288210)

Supplemental Material

Trends in cancer-free working life expectancy based on health insurance data from Germany – is the increase as strong as in working life expectancy?

Fabian Tetzlaff[1,2]*, Enno Nowossadeck[2], Jelena Epping[1,3], Vanessa di Lego[4,5], Magdalena Muszynska-Spielauer[4,5], Johannes Beller[1,3], Stefanie Sperlich[1], Juliane Tetzlaff[1]

[1] Medical Sociology Unit, Hannover Medical School, Hanover, Germany

[2] Division of Social Determinants of Health, Robert Koch-Institute, Berlin, Germany

[3] Comprehensive Cancer Center Hannover, Hannover Medical School, Hanover, Germany

[4] Wittgenstein Centre for Demography and Global Human Capital (IIASA, OeAW, Univ. Vienna), Vienna, Austria

[5] Vienna Institute of Demography, Austrian Academy of Sciences, Vienna, Austria

*Corresponding author

E-mail: TetzlaffF@rki.de

Table S1 Characteristics of the study population aged 18 to 69: exposures in person-years and number of failures by type of transition and time period, as well as educational group in the period 2011-2013

		Total				
		**2006-2008**	**2011-2013**	**2016-2018**	**lower educational level (2011-2013)**	**higher educational level (2011-2013)**
**Labour force**	number of individuals	1,115,220 (49%)	1,336,607 (53%)	1,691,937 (59%)	859,431 (69%)	162,582 (63%)
**Non-labour force**	number of individuals	1,165,388 (51%)	1,198,610 (47%)	1,154,517 (41%)	392,089 (31%)	95,190 (37%)
**Transition t_{12}** cancer-free labour force → cancer-free non-labour force	events	162,736 (91%)	200,516 (91%)	244,850 (91%)	129,019 (92%)	28,945 (96.5%)
	person years	2,500,447 (32%)	3,040,047 (35%)	3,717,477 (37%)	2,033,926 (42%)	334,681 (40%)
**Transition t_{13}** cancer-free labour force → cancer incident individuals	events	11,411 (6%)	14,626 (7%)	16,301 (6%)	8,883 (6%)	914 (3%)
	person years	2,561,940 (34%)	3,125,111 (35%)	3,838,131 (39%)	2,098,129 (44%)	346,970 (42%)
**Transition t_{14}** cancer-free labour force → deceased individuals	events	4,892 (3%)	5,542 (2%)	6,909 (3%)	2,440 (2%)	127 (0.5%)
	person years	2,629,210 (34%)	2,638,230 (30%)	2,392,656 (24%)	688,926 (14%)	153,815 (18%)
**Transition t_{21}** cancer-free non-labour force → cancer-free labour force	events	174,364 (61%)	211,222 (65%)	236,562 (68%)	140,242 (95%)	44,235 (99%)
	person years	2,629,210 (32%)	2,638,230 (32%)	2,392,656 (32%)	688,926 (32%)	153,815 (32%)
**Transition t_{23}** cancer-free non-labour force → cancer incidence	events	47,266 (17%)	46,830 (14%)	41,049 (12%)	5,553 (4%)	304 (0.7%)
	person years	2,682,165 (34%)	2,704,841 (34%)	2,456,404 (34%)	732,257 (34%)	168,242 (34%)
**Transition t_{24}** cancer-free non-labour force → deceased individuals	events	64,156 (22%)	68,930 (21%)	70,410 (20%)	2,580 (1%)	84 (0.3%)
	person years	2,682,096 (34%)	2,704,755 (34%)	2,456,318 (34%)	732,230 (34%)	168,242 (34%)

Data: Health insurance data from the AOKN (Allgemeine Ortskrankenkasse Niedersachsen)

Note: Education: lower education (less than 12 years of schooling) higher education (more than 12 years of schooling); Transitions: Cancer-free working life expectancy represents the expected number of years spent in the labour market that are free of cancer. Cancer-free working life expectancy is calculated based on the age-specific transition rates between four states: healthy labour force, healthy non-labour force, individuals with cancer, and death

Fig.S1 Time Trend in cancer incidence by labour force participation and sex (95%-CI)

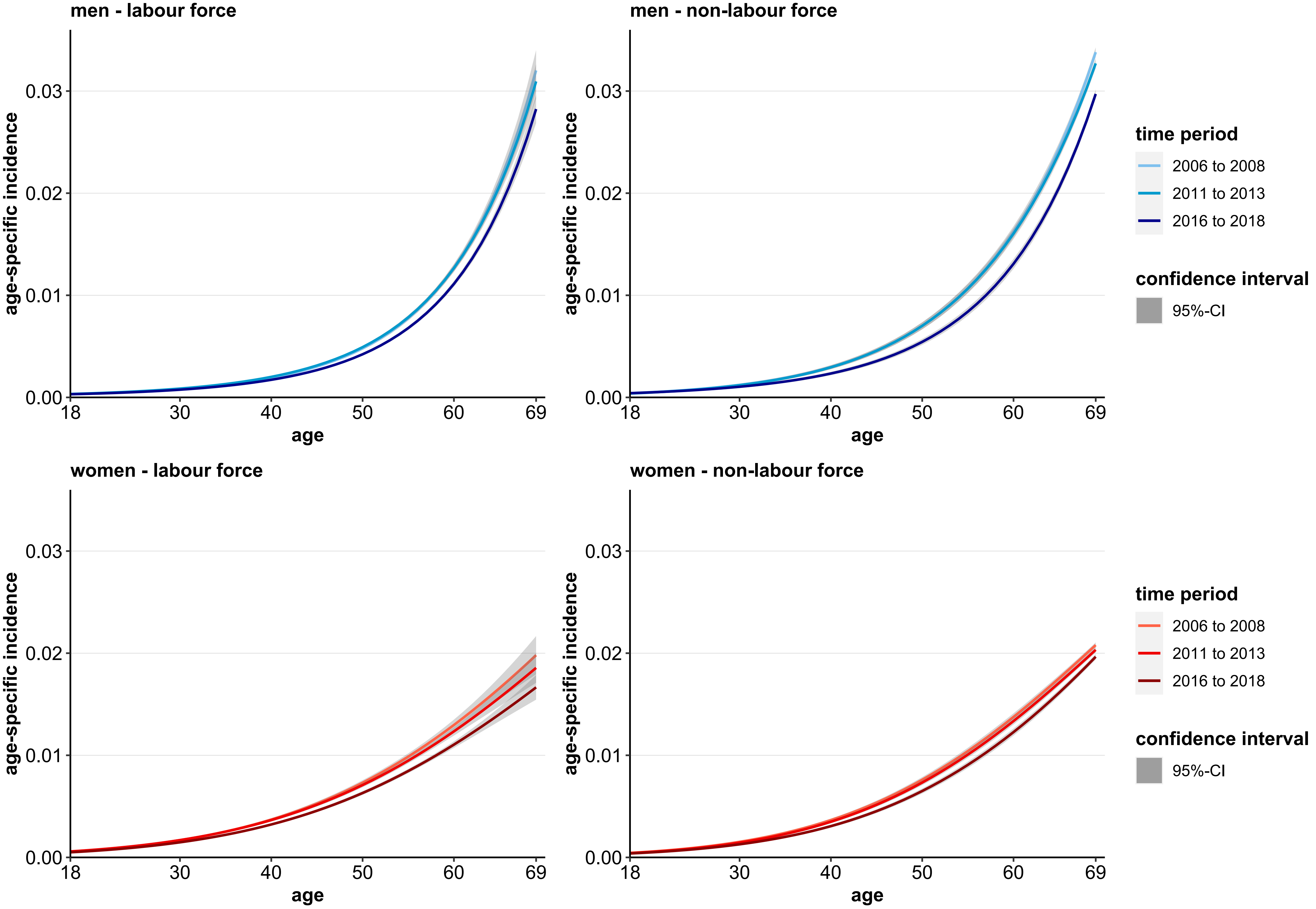

Fig.S2 Cancer incdence in labour force and non-labour force by sex and time period (95%-CI)

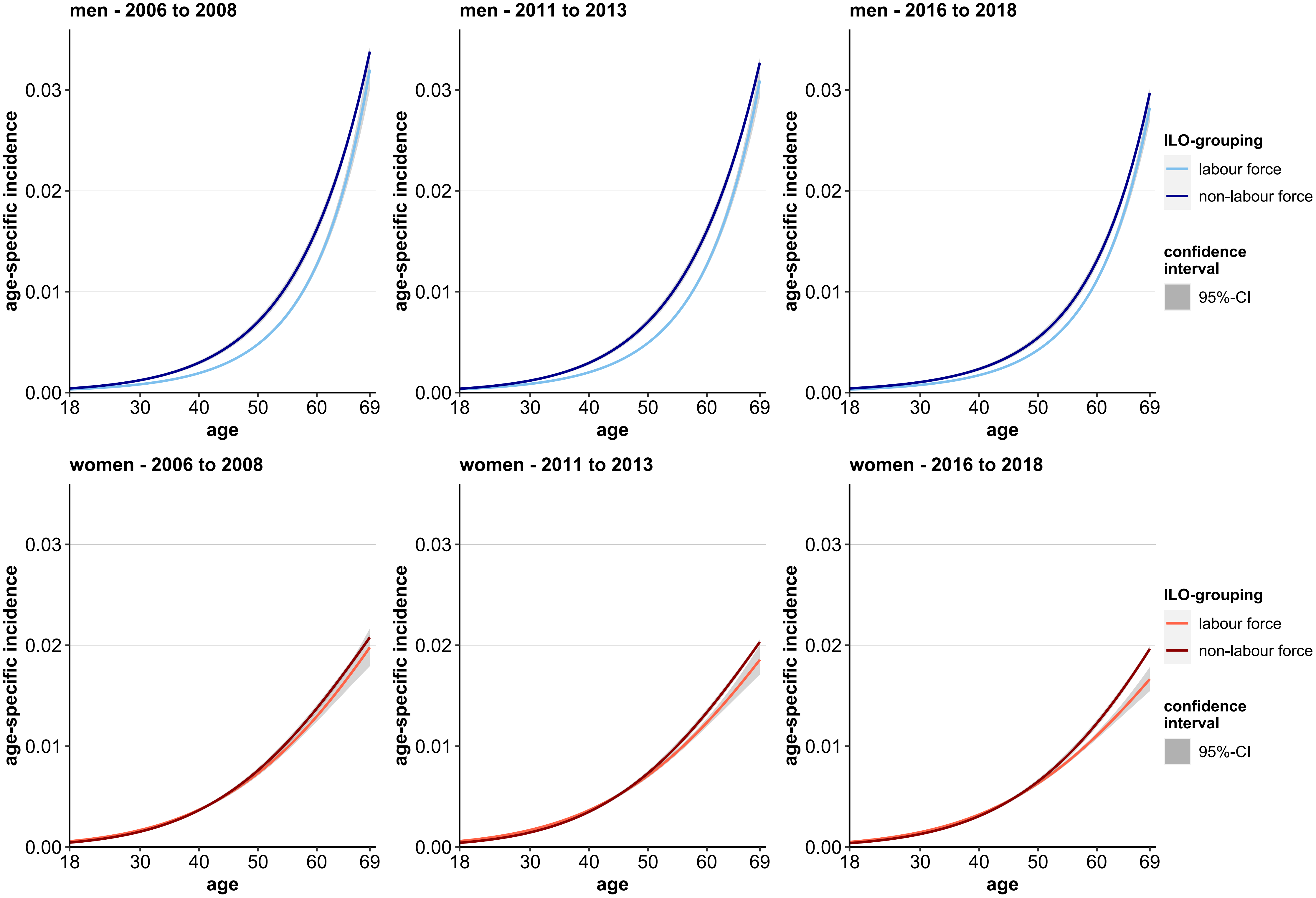

Fig.S3 Proportion cancer-free labour force in the total population (labour force plus non-labour force) across age by sex and period

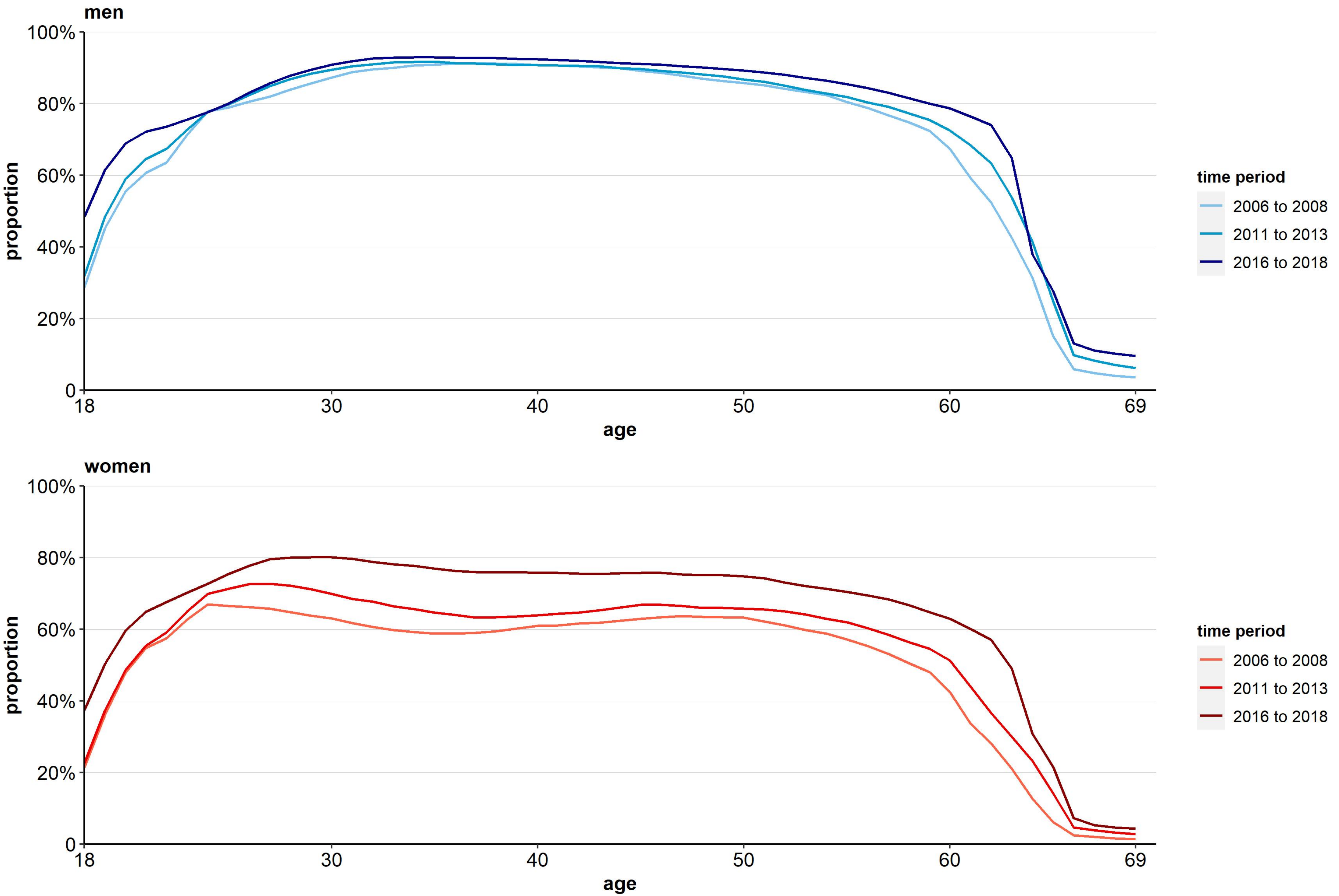

Fig.S4 Time trend in working and non-working life expectancy without cancer by sex (95%-CI)

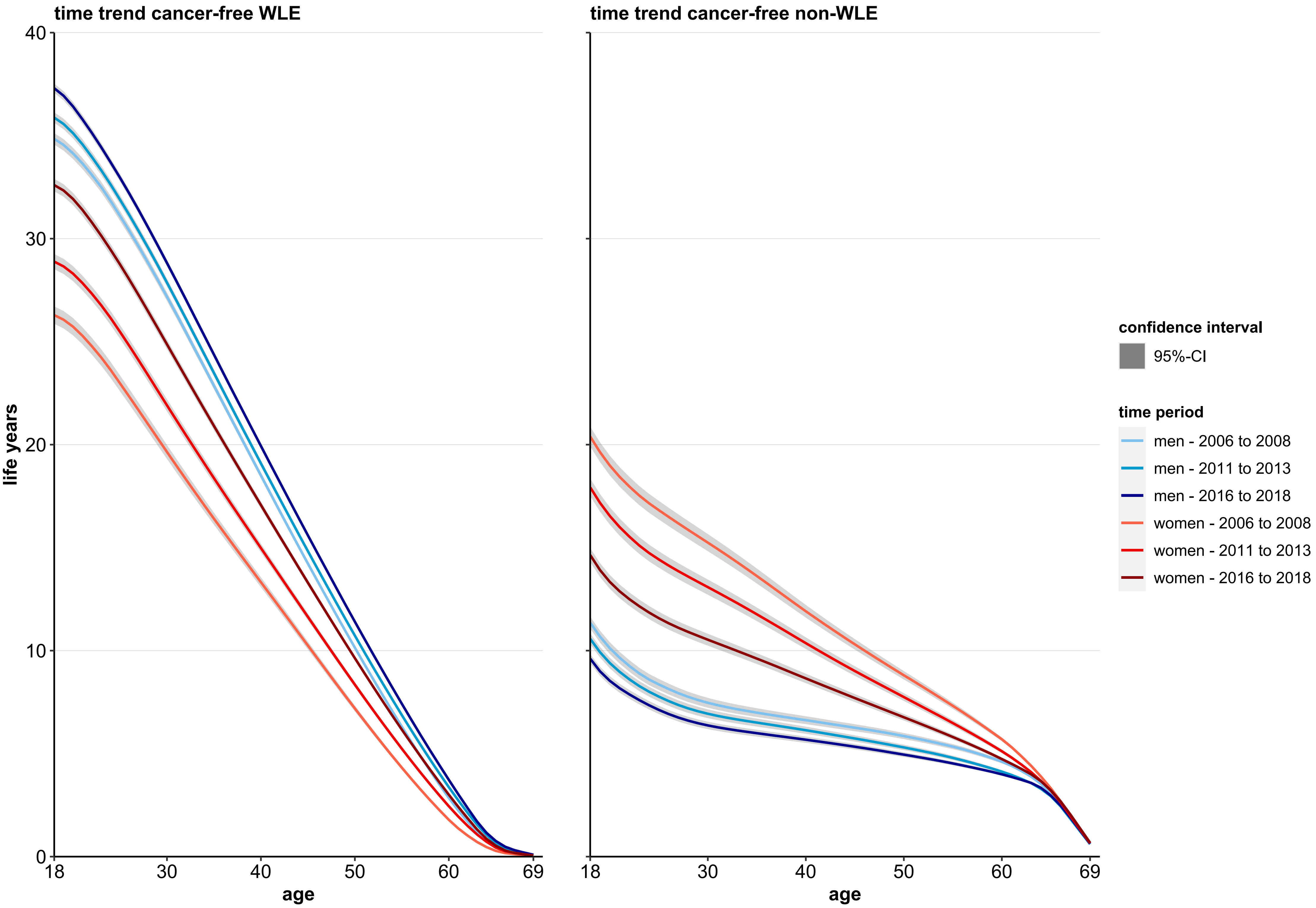

Supplement: S1 File — (PDF) [file pone.0288210.s001.pdf]
